# The complete chloroplast genome sequence of *Quercus franchetii* Skan (Fagaceae)

**DOI:** 10.1080/23802359.2021.1975515

**Published:** 2021-09-17

**Authors:** Ke-Nan Chen, Xiao-Long Jiang, Rong Yi

**Affiliations:** The Laboratory of Forestry Genetics, Central South University of Forestry and Technology, Changsha, China

**Keywords:** *Quercus franchetii*, Fagaceae, complete chloroplast genome

## Abstract

*Quercus franchetii* Skan, a crucial indicator plant of dry-hot valley with endemic to southwestern China. In this study, the complete chloroplast genome of *Q. franchetii* was assembled and characterized. The circular genome was 160,785 bp in length, containing a large single copy (LSC) region of 90,169 bp, a small single copy (SSC) region of 18,828 bp, and a pair of inverted repeat regions of 25,894 bp. Total 131 genes were annotated, comprising 86 protein-coding genes, 37 tRNA genes, and 8 rRNA genes. The phylogenetic analysis indicated that *Q. franchetii* was closely related to *Q. glauca* and *Q. chungii.*

*Quercus franchetii* Skan belongs to the *Quercus* section *Ilex* in the family Fagaceae. It is an evergreen broadleaf tree species endemic to southwestern China, which mainly distributed in Yunnan and Sichuan Provinces, with elevations ranging from 800 to 2600 m (Wu 1980). It is a key indicator plant in dry-hot valley of Jinsha River, one of the main constructive species in the karst mountains of Yunnan, and of great importance in ecological management of rocky desertification and dry-hot valleys (Liu et al. 2011, Chang et al. 2019). In spite of its important role in the ecology, molecular information for this species is limited. Here, we sequenced, assembled and annotated the complete chloroplast (CP) genome of *Q. franchetii* to contribute to its phylogenetic relationships with related species in the Fagaceae and serve as a resource for future genomic and genetic studies.

Fresh leaves of *Q*. *franchetii* were collected from Yuanjiang Hani, Yi and Dai Autonomous County, Yunnan province, China (23°34′01″N, 102°05′02″E, 994 m). The specimen was stored in Herbarium of Shanghai Chenshan Botanical Garden (CSH, http://csh.ibiodiversity.net/default.html, Bin-Jie GE, gebinjie123@163.com,) under the accession number DM19028. Total genomic DNA was extracted from silica-dried leaves using DNeasy plant tissue kit (TIANGEN Biotech Co., Ltd., Beijing, China) and sequenced on Illumina Hiseq X Ten platform. A total of 70,147,322 clean reads were generated and 50,000,000 were used to assemble the complete chloroplast genomes by GetOrganelle v1.7.2beta (Jin et al. 2020) . Annotation was executed using PGA pipeline (Qu et al. 2019). The chloroplast genome together with gene annotations was submitted to GenBank with accession number MW450869.

The complete chloroplast genome of *Q*. *franchetii* was 160,785 bp in size with a typical quadripartite circular structure, which was composed of a large single copy region (LSC), a small single copy region (SSC), and two inverted repeats (IRs) with 90,169, 18,828, and 25,894 bp, respectively. The GC content of whole chloroplast DNA was 36.93%, while the LSC, SSC, and the IR regions were 34.8, 31.13, and 42.71%, respectively. The chloroplast genome contained 131 genes, including 86 protein-coding genes, 37 tRNA genes, and 8 rRNA genes.

To investigate the phylogenetic position of *Q*. *franchetii* within the *Quercus*, a total of 15 complete chloroplast genomes of Fagaceae were obtained from GenBank, with *Lithocarpus hancei* (Bentham) Rehd. and *Castanopsis fargesii* Franch. as outgroups. The 16 complete chloroplast sequences were aligned by the MAFFT v7.475 software (Katoh and Standley 2013). Phylogenetic tree was reconstructed using maximum-likelihood (ML) analysis implemented in IQ-TREE v1.6.12 with 1000 bootstrap replicates (Nguyen et al. 2015). The results indicated that *Q. franchetii* was mostly related to *Q. glauca* and *Q. chungii* with strong bootstrap support (Figure 1).

**Figure 1.**
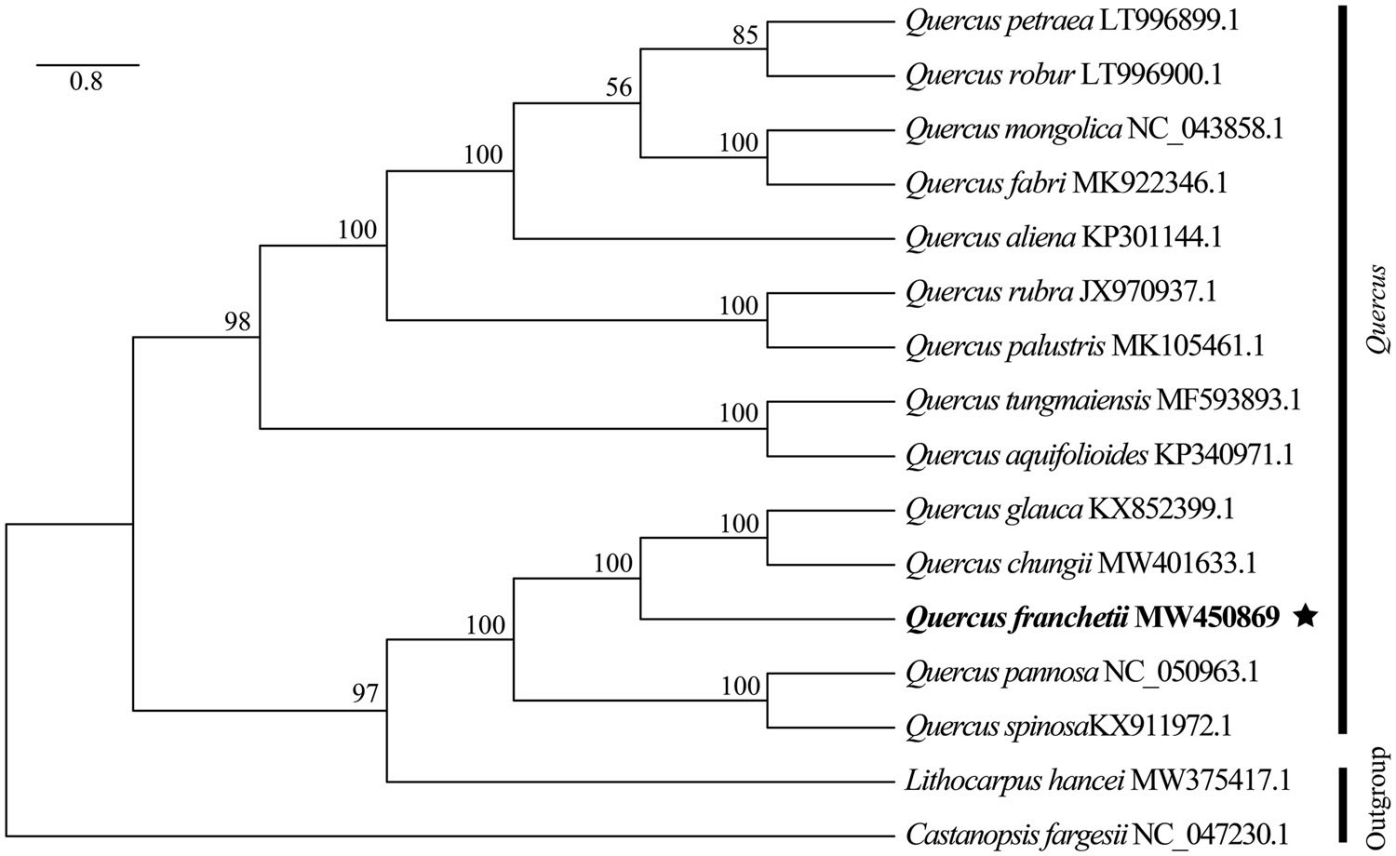
Phylogenetic tree based on 16 complete chloroplast genome sequences of Fagaceae. The bootstrap support values are indicated in the nodes.

## Data Availability

The complete chloroplast genome sequence of *Quercus franchetii* is deposited in the GenBank database under the accession number MW450869 (https://www.ncbi.nlm.nih.gov/nuccore/MW450869). The associated BioProject, SRA, and Bio-Sample numbers are PRJNA725598, SRR14338619, and SAMN18893589, respectively.
